# Pneumococcal vaccination coverage and adherence to recommended dosing schedules in adults: a repeated cross-sectional study of the INTEGO morbidity registry

**DOI:** 10.1186/s12889-023-15939-7

**Published:** 2023-06-07

**Authors:** Arne Janssens, Bert Vaes, Chloé Abels, Jonas Crèvecoeur, Pavlos Mamouris, Barbara Merckx, Pieter Libin, Gijs Van Pottelbergh, Thomas Neyens

**Affiliations:** 1 Department of Public Health and Primary Care, Faculty of Medicine, Academic Centre of General Practice, KU Leuven, Kapucijnenvoer 35, Leuven, B-3000 Belgium; 2 MSD, Brussels, Belgium; 3 I-BioStat, Data Science Institute, Hasselt University, Martelarenlaan 42, B-3500 Hasselt, Belgium; 4 Interuniversity Institute of Biostatistics and Statistical Bioinformatics, Data Science Institute, Hasselt University, Hasselt, Belgium; 5 Artificial Intelligence Lab, Department of Computer Science, Vrije Universiteit Brussel, Brussels, Belgium; 6 Department of Microbiology and Immunology, Rega Institute for Medical Research, Clinical and Epidemiological Virology, KU Leuven, Leuven, Belgium; 7 Department of Public Health and Primary Care, Faculty of Medicine, L-BioStat, KU Leuven, Kapucijnenvoer 35, Leuven, B-3000 Belgium

**Keywords:** Adult pneumococcal vaccination, Vaccination recommendations, Vaccination schedule adherence, General practice, Equity, Generalized linear model, Logistic regression

## Abstract

**Background:**

Since 2014, Belgium’s Superior Health Council has recommended pneumococcal vaccination for adults aged 19–85 years at increased risk for pneumococcal diseases with a specific vaccine administration sequence and timing. Currently, Belgium has no publicly funded adult pneumococcal vaccination program. This study investigated the seasonal pneumococcal vaccination trends, evolution of vaccination coverage and adherence to the 2014 recommendations.

**Methods:**

INTEGO is a general practice morbidity registry in Flanders (Belgium) that represents 102 general practice centres and comprised over 300.000 patients in 2021. A repeated cross-sectional study was performed for the period between 2017 and 2021. Using adjusted odds ratios computed via multiple logistic regression, the association between an individual’s characteristics (gender, age, comorbidities, influenza vaccination status and socioeconomic status) and schedule-adherent pneumococcal vaccination status was assessed.

**Results:**

Pneumococcal vaccination coincided with seasonal flu vaccination. The vaccination coverage in the population at risk decreased from 21% in 2017 to 18.2% in 2018 and then started to increase to 23.6% in 2021. Coverage in 2021 was highest for high-risk adults (33.8%) followed by 50- to 85-year-olds with comorbidities (25.5%) and healthy 65- to 85-year-olds (18.7%). In 2021, 56.3% of the high-risk adults, 74.6% of the 50+ with comorbidities persons, and 74% of the 65+ healthy persons had an adherent vaccination schedule. Persons with a lower socioeconomic status had an adjusted odds ratio of 0.92 (95% Confidence Interval (CI) 0.87–0.97) for primary vaccination, 0.67 (95% CI 0.60–0.75) for adherence to the recommended second vaccination if the 13-valent pneumococcal conjugate vaccine was administered first and 0.86 (95% CI 0.76–0.97) if the 23-valent pneumococcal polysaccharide vaccine was administered first.

**Conclusion:**

Pneumococcal vaccine coverage is slowly increasing in Flanders, displaying seasonal peaks in sync with influenza vaccination campaigns. However, with less than one-fourth of the target population vaccinated, less than 60% high-risk and approximately 74% of 50 + with comorbidities and 65+ healthy persons with an adherent schedule, there is still much room for improvement. Furthermore, adults with poor socioeconomic status had lower odds of primary vaccination and schedule adherence, demonstrating the need for a publicly funded program in Belgium to ensure equitable access.

**Supplementary Information:**

The online version contains supplementary material available at 10.1186/s12889-023-15939-7.

## Background

In Europe, *Streptococcus pneumoniae*(SP) is the most prevalent bacterial agent in lower respiratory tract infections (LRTIs) and the most common cause of community-acquired pneumonia [1, 2]. SP is responsible for approximately 20% of all fatal LRTI cases, accounting for more than one million deaths annually; therefore, SP can be qualified as one of the most lethal pathogens [2].

Until recently, two pneumococcal vaccines were available for adults in Europe: the 23-valent pneumococcal polysaccharide vaccine (PPSV23) and the 13-valent pneumococcal conjugate vaccine (PCV13), which protect against 23 and 13 different serotypes of the bacterium *Streptococcus pneumoniae*, respectively. Two new vaccines, the 15-valent and 20-valent pneumococcal conjugate vaccines, were recently approved by the European Medicines Agency [3, 4]. Typically, adult pneumococcal vaccination is recommended for older and immunocompromised adults by means of specific recommendations of vaccine sequence and timing. However, national guidelines for pneumococcal vaccination differ between countries, depend on public reimbursements of these vaccines, and include age-based and/or risk-based strategies [5, 6]. Most European countries have implemented age-based vaccination programs. The Vaccine Scheduler developed by The European Centre for Disease Prevention and Control provides an age-based overview of recommended vaccination schedules in 30 European countries [7].

Between 1993 and 2014, the Belgian Superior Health Council, an advisory body that forms a bridge between Belgian authorities and the scientific world in all public health-related issues, recommended vaccination with PPSV23 for all adults aged 60 years or older [8]. Since 2014, the Superior Health Council recommends pneumococcal vaccination for three target groups consisting of adults with an increased risk of pneumococcal disease: immunocompromised adults (19–85 years old at high risk, referred to here as high-risk persons), adults aged 50–85 years with comorbidity (50+ with comorbidity) and healthy adults aged 65 to 85 (65+ healthy) [9]. The recommendations include a primary vaccination that consists of a sequential scheme of PCV13 followed by PPSV23 at least eight weeks later or a primary one-time PCV13 vaccine a year after the last PPSV23 and a revaccination with PPSV23 every five years for high-risk adults. The Belgian pneumococcal vaccination recommendations were updated in 2020 to include an alternative scheme of PPSV23 alone in healthy adults aged 65 to 85, two new risk groups (adults with diabetes and adults with chronic neurological or neuromuscular disorders with aspiration risk), and a revaccination with PPSV23 after 5 years for adults aged 50–85 years with comorbidities [10]. Very recent, in September 2022, the Belgian recommendations were revised and include the newly approved PCV20 and PCV15 vaccines [11].

Pneumococcal vaccination recommendations are introduced to ensure an optimal protection of risk groups against pneumococcal diseases. Vaccination with PCV13 and PPSV23 showed protective effects for at-risk adults against severe LRTIs [12]. However, only a few studies have addressed the adherence of adult pneumococcal vaccination to these recommendations. One study conducted in the United States showed that even after six years of follow-up, only 14.2% of high-risk adults received any pneumococcal vaccination, and only 2.2% completed the pneumococcal vaccination sequence [13]. In Belgium, pharmacists reported that during the period between 2014 and 2017, approximately 41% of adults receiving a PCV13 vaccine were compliant with the vaccination scheme [14].

Pneumococcal vaccination coverage in the at-risk adult population in Belgium in 2015, one year after the updates in the 2014 recommendations, was only 19%, and was higher in older adults and those at risk [15]. It is known that implementation and adoption of new guidelines takes time [16]. This is especially the case if an implementation strategy addressing the barriers to guideline adoption is lacking [16]. One of the main barriers to adult pneumococcal vaccination in Belgium – in contrast to most other countries in Europe – is the lack of a publicly funded vaccination program.

Therefore, we aimed to investigate the evolution of pneumococcal vaccination coverage and schedule adherence in Flanders after the introduction of the 2014 recommendations and to identify individual characteristics affecting pneumococcal vaccination and adherence to recommended vaccination schedules.

## Methods

### Study population and design

The INTEGO network is a morbidity registry containing coded contents of electronic health records from over 100 general practice centres all over Flanders (Belgium). In 2021, INTEGO comprised approximately 340.000 patients, covering 5.7% of the Flemish population. The information is automatically collected during daily practice and contains diagnoses, sociodemographic information (i.e., year of birth, gender, and an indicator denoting increased reimbursement as proxy for socioeconomic status), prescriptions, vaccination status, laboratory results and various biomedical parameters, such as blood pressure, height, weight, etc. The INTEGO procedures have been validated by the Belgian Privacy Commission [17, 18]. The registry is hosted on the Healthdata platform (www.healthdata.be) [18]. In 2017, INTEGO practices migrated from the medical software Medidoc® to CareConnect® (Corilus, Gent, Belgium) [18]. Consequently, there are two databases: the old database (before 2018) and the new database (from 2018 onwards).

The study cohort comprised all persons between 19 and 85 years old with an electronic medical record in one of the general practices from the INTEGO network during the period of 2017–2021. Only good registering practices were selected for the current study. A practice that coded at least 80% of their registered diagnoses (International Classification of Primary Care version 2 (ICPC-2)) was considered a good registrant. This condition was satisfied by 86 INTEGO practices.

For each year in the period 2017–2021, the yearly contact group (YCG), which consists of individuals with at least one general practitioner appointment in a certain year, was constructed. Due to the transition of INTEGO in 2017–2018, records from new practices only contribute to the YCGs from 2018 onwards, and records from old practices contribute to all YCGs.

### Outcome definition

#### Pneumococcal vaccination coverage and primary vaccination

We computed monthly vaccination rate, yearly vaccination coverage and yearly rate of primary vaccination for each of the three target groups and the total population at risk, as defined by the vaccination recommendations. The monthly vaccination rate was computed as the proportion of persons receiving a vaccine (PCV13 or PPSV23) in a certain month with respect to the number of persons in the corresponding YCG. The yearly vaccination coverage was defined as the proportion of persons in the corresponding YCG who received a pneumococcal vaccination (PCV23 or PPSV23) before or in the given year. Primary vaccination was the first adult pneumococcal vaccination, i.e., the first vaccination with PCV13 or PPSV23 at an age of 19 or older. The yearly rate of primary vaccination for a given target group and a given year was then defined as the fraction of persons in the target group of the YCG who were never vaccinated before and who received their first dose by the end of the given year. These three measures were used for summary statistics. The latter also provided an identification of individuals who received their first pneumococcal vaccine in a given year. The resulting dataset contained multiple records for the same person describing different years as long as this person was not vaccinated, i.e., the person did not receive a pneumococcal vaccination after the age of 19. After vaccination, persons were excluded from the study population for future years. Then, we used this dataset to investigate the potential association between primary vaccination and different person-related factors by modelling the probability of primary vaccination as a function of these factors.

#### Overall schedule adherence and adherence to the second vaccine

To explore overall vaccination schedule adherence, we computed the proportion of persons in the considered target group of the YCG of each year with a vaccination profile according to the recommendations. To this end, we implemented the algorithm given in Table 1. This algorithm approximates the recommendations and determines for a given target group and a given year if an individual had a vaccination profile, i.e., all registered pneumococcal vaccines until the end of the given year, in adherence to the recommended schedule.

**Table 1:** The algorithm to determine overall schedule adherence in a given year

	**Vaccination profile of an individual**			
**Target group**	**who received only one type of vaccine**		**who received both types of vaccines**	
	**PCV13**	**PPSV23**	**First vaccine: PCV13**	**First vaccine: PPSV23**
High-risk	last pcv13 less than 1 year ago	last ppsv23 less than 5 years ago and if multiple ppsv23 doses, a TI_ppsv23_ between 4 and 6 years	followed by a ppsv23 after at least 8 weeks and the last ppsv23 less than 5 years ago and if multiple ppsv23 doses, a TI_ppsv23_ between 4 and 6 years	followed by a pcv13 after at least 1 year and the last ppsv23 less than 5 years ago and if multiple ppsv23 doses, a TI_ppsv23_ between 4 and 6 years
50+ with comorbidity	last pcv13 less than 1 year ago	last ppsv23 less than 5 years ago	followed by a ppsv23 after at least 8 weeks	followed by a pcv13 after at least 1 year
65+ healthy	last pcv13 less than 1 year ago	last ppvs23 less than 5 years ago	followed by a ppsv23 after at least 8 weeks	followed by a pcv13 after at least 1 year

TI_psv23_ represents the median time interval between subsequent ppsv23 vaccines

To investigate the effect of an individual’s characteristics on schedule adherence, we modelled the probability of receiving a second vaccine in line with the recommendations. At the moment of the first vaccination, it was unknown whether a person would complete the remainder of the recommended vaccination schedule. With a PCV13 as the first vaccine, it was recommended to receive the subsequent PPSV23 dose after at least eight weeks and with a PPSV23 as the first vaccine, it was recommended to receive a subsequent PCV13 vaccine after at least one year or a revaccination with a PPSV23 dose after approximately five years. For modelling purposes and based on expert knowledge, we introduced a time interval within which the person should receive their second vaccine to adhere to the recommendations. We considered two groups:

- Group 1 included persons who received PCV13 as the first vaccine and should receive a PPSV23 dose between 8 weeks and one year later, to adhere to the recommendations.
- Group 2 included persons who received PPSV23 as the first vaccine and should receive the PCV13 dose after at least one year and a maximum of six years or a second PPSV23 dose after at least four years and a maximum of six years to adhere to the recommendations.

### Covariates

To investigate the association between an individual’s characteristics and the two vaccination outcomes, i.e., primary vaccination and adherence to second vaccine, we constructed the following covariates: (i) gender (female, male), (ii) age groups (19–49, 50–64, and 65–85 years of age), (iii) risk groups (low, medium, and high) based on comorbidities determined by diagnoses, prescriptions and lab data as given in Additional file 1, (iv) target group that combines age and risk covariates according to the recommendations (high risk, 50+ with comorbidity, 65+ healthy, and nontarget), (v) influenza vaccination status (yes, no), (vi) receiving increased reimbursement of healthcare (yes, no, and unknown) as a proxy for socioeconomic status, (vii) smoking status (smoker, ex-smoker, and never smoker) that was longitudinally imputed (see Additional file 2 for a brief explanation), and (viii) the year of measurement for the primary vaccination outcome and the year of first vaccination for the schedule adherence outcome. All covariates were determined in the year of measurement for primary vaccination and the year of first vaccination for schedule adherence. The values of socioeconomic status and smoking, which were only available from 2016 onwards, were approximated based on its closest-in-time value with respect to the year of measurement.

### Statistical analysis

#### General

To investigate the effect of the person characteristics on the outcomes, adjusted odds ratios (aORs) and their 95% confidence intervals (CIs) were computed using multiple logistic regression. The reported aOR estimates were pooled estimates, applying Rubin’s rules (see Additional file 2), over the 20 imputed datasets. Trends of vaccination coverage over the period of 2017–2021 were explored with the Cochran-Armitage test using year as an ordinal variable and vaccination status (i.e., did the individual ever receive a vaccination) as the binary outcome variable. All analyses were performed using R [19] Software V4.0.3 (*DescTool, stats* and *mice* packages).

#### Model outcome 1: Primary vaccination

For a person $i$ in a certain year, the outcome ${Y}_{i}^{vaccinated}$, which was 1 if the person was vaccinated in that given year and 0 otherwise, was assumed to follow a Bernoulli distribution:

$${Y}_{i}^{vaccinated} \sim Bernouilli\left({p}_{i}^{vaccinated}\right)$$

The logit of the probability of primary vaccination ${p}_{i}^{vaccinated}$ was modelled as follows:

$$\begin{array}{l}logit\left({p}_{i}^{vaccinated}\right)=\mathrm{log}\left({}^{{p}_{i}^{vaccinated}}\!\left/ \!{}_{1-{p}_{i}^{vaccinated}}\right.\right)\\ ={\beta }_{0}+{\varvec{\beta}}\boldsymbol{*}{{\varvec{x}}}_{{\varvec{i}}}\\ ={\beta }_{0}+{\beta }_{1}*{gender}_{{female}_{i}}+{\beta }_{2}*{influenza\_vaccination}_{{yes}_{i}}+\\ +{\beta }_{3}*{increased\_reimbursement}_{{yes}_{i}}+\\ +{\beta }_{4}*{increased\_reimbursement}_{{unknown}_{i}}+\\ +{\beta }_{5} * {smoking}_{{ex\_smoker}_{i}}+{\beta }_{6}*{smoking}_{{smoker}_{i}}+\\ +{\beta }_{7}*{target}_{{high\_risk}_{i}}+{\beta }_{8}*{target}_{{50+\_with\_comorbidity}_{i}}+\\ +{\beta }_{9}* {target}_{{65+\_healthy}_{i}}+\\ +{\beta }_{10}*{2018}_{i}+\dots +{\beta }_{13}*{2021}_{i}\end{array}$$

where ${{\varvec{x}}}_{{\varvec{i}}}$ represents the vector of covariates and ${\varvec{\beta}}$ the vector of corresponding regression coefficients. The year refers to the year that the outcome was measured.

#### Model outcome 2: Adherence to recommended second vaccination

Two scenarios were considered. There was a group of persons with PCV13 as the first vaccine and a group with PPSV23 as the first vaccine. The outcomes (and corresponding probabilities) are ${Y}_{i}^{adherence\_pcv13}$ (${p}_{i}^{adherence\_pcv13}$) and ${Y}_{i}^{adherence\_ppsv23}$ (${p}_{i}^{adherence\_ppsv23}$), respectively. Both were modelled in a similar fashion:

$${Y}_{i}^{adherence} \sim Bernoulli\left({p}_{i}^{adherence}\right)$$

With the logit of the probability of receiving a second vaccine in adherence to the recommendations ${p}_{i}^{adherence}$ modelled as:

$$\begin{array}{l}logit\left({p}_{i}^{adherence}\right)=\mathrm{log}\left({}^{{p}_{i}^{adherence}}\!\left/ \!{}_{1-{p}_{i}^{adherence}}\right.\right)\\ ={\beta }_{0}+{\varvec{\beta}}\boldsymbol{*}{{\varvec{x}}}_{{\varvec{i}}}\\ ={\beta }_{0}+{\beta }_{1}*{gender}_{{female}_{i}}+{\beta }_{2}*{influenza\_vaccination}_{{yes}_{i}}+\\ +{\beta }_{3}*{increased\_reimbursement}_{{yes}_{i}}+\\ +{\beta }_{4}*{increased\_reimbursement}_{{unknown}_{i}}+\\ +{\beta }_{5} * {smoking}_{{ex\_smoker}_{i}}+{\beta }_{6}*{smoking}_{{smoker}_{i}}+\\ +{\beta }_{7}*{target}_{{high\_risk}_{i}}+{\beta }_{8}*{target}_{{50+\_with\_comorbidity}_{i}}+\\ +{\beta }_{9}* {target}_{{65+\_healthy}_{i}}+\\ +{\beta }_{10}*{1997}_{i}+\dots +{\beta }_{28}*{2015}_{i}\end{array}$$

where ${{\varvec{x}}}_{{\varvec{i}}}$ represents the vector of covariates and ${\varvec{\beta}}$ the vector of corresponding regression coefficients. The year was the year of first vaccination.

## Results

### Study population

#### Yearly contact group structure

Table 2 shows the YCG population in terms of risk groups as defined by the 2014 recommendations. The number of persons with a certain characteristic and their proportion with respect to the top-level total are given as n (%). For the imputed smoking characteristic, the median, quartile 1 (Q1) and quartile 3 (Q3) of the number and proportion of persons is given. In the period of 2017–2021, on average, 29.5% of the adult population in the YCG was at risk of pneumococcal disease and belonged to the target group of vaccination (2.7% high risk, 15.2% 50+ with comorbidity, 11.6% 65+ without comorbidity). The number of persons doubled between 2017 and 2018 due to the expansion of the INTEGO network.

**Table 2:** Yearly contact group structure in terms of individual characteristics

Yearly contact group population characteristics (n (%) and median [Q1-Q3])					
**Year**	**2017**	**2018**	**2019**	**2020**	**2021**
**Total population**	79,645	185,815	194,355	201,995	214,147
**High-risk group**	2253 (2.8)	4791 (2.6)	5315 (2.7)	5638 (2.8)	5635 (2.6)
Immunized	612 (27.2)	1239 (25.9)	1539 (29)	1845 (32.7)	1902 (33.8)
Adherence	326 (53.3)	698 (56.3)	888 (57.7)	1130 (61.2)	1070 (56.3)
Female	1200 (53.3)	2548 (53.2)	2819 (53)	2998 (53.2)	2958 (52.5)
Influenza vaccination	846 (37.5)	1856 (38.7)	2093 (39.4)	2415 (42.8)	1966 (34.9)
Increased reimbursement	463 (20.6)	1088 (22.7)	1188 (22.4)	1181 (20.9)	1204 (21.4)
Smoking					
Ex-smoker	650 [638–666] (29 [28-30])	1574 [1552–1605] (33 [32-34])	1882 [1858–1914] (35 [35-36])	2084 [2069–2102] (37 [37-37])	2180 [2166–2217] (39 [38-39])
Never smoker	1064 [1057–1086] (47 [47-48])	2185 [2161–2192] (46 [45-46])	2313 [2288–2326] (44 [43-44])	2355 [2339–2378] (42 [41-42])	2318 [2291–2332] (41 [41-41])
Smoker	528 [520–539] (23 [23-24])	1029 [1017–1043] (22 [21-22])	1114 [1102–1144] (21 [21-22])	1204 [1176–1214] (21 [21-22])	1138 [1124–1164] (20 [20-21])
Number of high-risk comorbidities					
1	2107 (93.5)	4473 (93.4)	4983 (93.8)	5294 (93.9)	5287 (93.8)
≥ 2	146 (6.5)	318 (6.6)	332 (6.2)	344 (6.1)	348 (6.2)
Number of medium-risk comorbidities					
1	561 (24.9)	1186 (24.8)	1424 (26.8)	1476 (26.2)	1468 (26.1)
2	258 (11.5)	503 (10.5)	572 (10.8)	657 (11.7)	631 (11.2)
≥ 3	217 (9.6)	411 (8.6)	465 (8.7)	528 (9.4)	542 (9.6)
**50+ with comorbidity group**	13,101 (16.4)	26,534 (14.3)	28,888 (14.9)	31,123 (15.4)	32,406 (15.1)
Immunized	3003 (22.9)	5361 (20.2)	6353 (22)	7804 (25.1)	8252 (25.5)
Adherence	2138 (71.2)	3798 (70.8)	4663 (73.4)	5984 (76.7)	6158 (74.6)
Female	6435 (49.1)	12,915 (48.7)	14,168 (49)	15,307 (49.2)	16,026 (49.5)
Influenza vaccination	5915 (45.1)	11,462 (43.2)	12,543 (43.4)	15,171 (48.7)	12,654 (39)
Increased reimbursement	2908 (22.2)	5990 (22.6)	6273 (21.7)	6500 (20.9)	6617 (20.4)
Smoking					
Ex-smoker	4741 [4707–4766] (36 [36-36])	10,429 [10354–10462] (39 [39-39])	12,012 [11942–12066] (42 [41-42])	13,406 [13360–13512] (43 [43-43])	14,396 [14317–14454] (44 [44-45])
Never smoker	5610 [5566–5640] (43 [42-43])	10,720 [10633–10765] (40 [40-41])	11,214 [11181–11304] (39 [39-39])	11,762 [11700–11817] (38 [38-38])	11,888 [11852–11964] (37 [37-37])
Smoker	2760 [2734–2811] (21 [21-21])	5423 [5358–5457] (20 [20-21])	5660 [5609–5710] (20 [19-20])	5956 [5888–5990] (19 [19-19])	6133 [6042–6184] (19 [19-19])
Number of medium-risk comorbidities					
1	8152 (62.2)	17,411 (65.6)	18,630 (64.5)	19,794 (63.6)	20,500 (63.3)
2	2938 (22.4)	5701 (21.5)	6321 (21.9)	6897 (22.2)	7207 (22.2)
≥ 3	2011 (15.3)	3422 (12.9)	3937 (13.6)	4432 (14.2)	4699 (14.5)
**65+ healthy group**	9302 (11.7)	22,181 (11.9)	22,734 (11.7)	23,601 (11.7)	23,971 (11.2)
Immunized	1570 (16.9)	3122 (14.1)	3540 (15.6)	4417 (18.7)	4482 (18.7)
Adherence	1122 (71.5)	2201 (70.5)	2610 (73.7)	3445 (78)	3315 (74)
Female	5347 (57.5)	12,736 (57.4)	12,999 (57.2)	13,398 (56.8)	13,524 (56.4)
Influenza vaccination	3816 (41)	9297 (41.9)	9538 (42)	11,497 (48.7)	9105 (38)
Increased reimbursement	1909 (20.5)	4392 (19.8)	4295 (18.9)	4410 (18.7)	4247 (17.7)
Smoking					
Ex-smoker	3332 [3301–3364] (36 [36-36])	8508 [8464–8534] (38 [38-38])	9234 [9199–9264] (41 [40-41])	10,018 [9965–10042] (42 [42-43])	10,396 [10358–10464] (43 [43-44])
Never smoker	4290 [4275–4342] (46 [46-47])	10,134 [10106–10173] (46 [46-46])	10,088 [10030–10142] (44 [44-45])	10,095 [10063–10150] (43 [43-43])	10,024 [9995–10073] (42 [42-42])
Smoker	1668 [1656–1694] (18 [18-18])	3520 [3493–3602] (16 [16-16])	3401 [3351–3458] (15 [15-15])	3476 [3440–3512] (15 [15-15])	3520 [3484–3564] (15 [14-15])
**Nontarget group**	54,989 (69)	132,309 (71.2)	137,418 (70.7)	141,633 (70.1)	152,135 (71)
Immunized	535 (1)	1305 (1)	1507 (1.1)	1864 (1.3)	1893 (1.2)
Adherence					
Female	29,564 (53.8)	71,838 (54.3)	74,260 (54)	76,261 (53.8)	80,296 (52.8)
Influenza vaccination	3439 (6.3)	9030 (6.8)	9243 (6.7)	11,630 (8.2)	13,487 (8.9)
Increased reimbursement	6764 (12.3)	15,741 (11.9)	16,364 (11.9)	16,466 (11.6)	17,328 (11.4)
Smoking					
Ex-smoker	9886 [9820–9936] (18 [18-18])	28,960 [28776–29016] (22 [22-22])	33,654 [33604–33758] (24 [24-25])	37,889 [37595–38031] (27 [26-27])	43,288 [43027–43480] (28 [28-29])
Never smoker	29,624 [29513–29708] (54 [54–54])	68,640 [68527–68830] (52 [52–52])	68,904 [68780–68981] (50 [50–50])	68,567 [68396–68771] (48 [48-49])	71,310 [71093–71482] (47 [47-47])
Smoker	15,516 [15396–15632] (28 [28-28])	34,683 [34606–34784] (26 [26-26])	34,872 [34728–35014] (25 [25-26])	35,131 [35050–35256] (25 [25-25])	37,530 [37386–37770] (25 [25-25])
Number of medium-risk comorbidities					
1	4816 (8.8)	9603 (7.3)	10,571 (7.7)	11,329 (8)	12,327 (8.1)
2	443 (0.8)	802 (0.6)	883 (0.6)	1026 (0.7)	1156 (0.8)
≥ 3	106 (0.2)	160 (0.1)	168 (0.1)	178 (0.1)	192 (0.1)

The number of persons with a certain characteristic and the proportion with respect to the upper-level population is given as n (%)

Smoking: Over 20 imputed datasets, the median [Q1-Q3] of the number and proportion of persons

Greater than half of the persons in the high-risk and 65+ healthy groups were women, and this distribution was similar for each YCG. Approximately one-fifth of the persons in each target group received increased compensation, i.e., higher reimbursement for medical costs. Furthermore, in each year, one-quarter of the persons at high risk had one medium-risk comorbidity in addition to at least one high-risk comorbidity. Table 2 also captures the influenza and pneumococcal vaccination coverage in each year. Additional file 3 shows the 2019 population distribution according to age and risk (i.e., comorbidities) groups.

#### Study population for primary vaccination and adherence to second vaccine

**Fig. 1:**
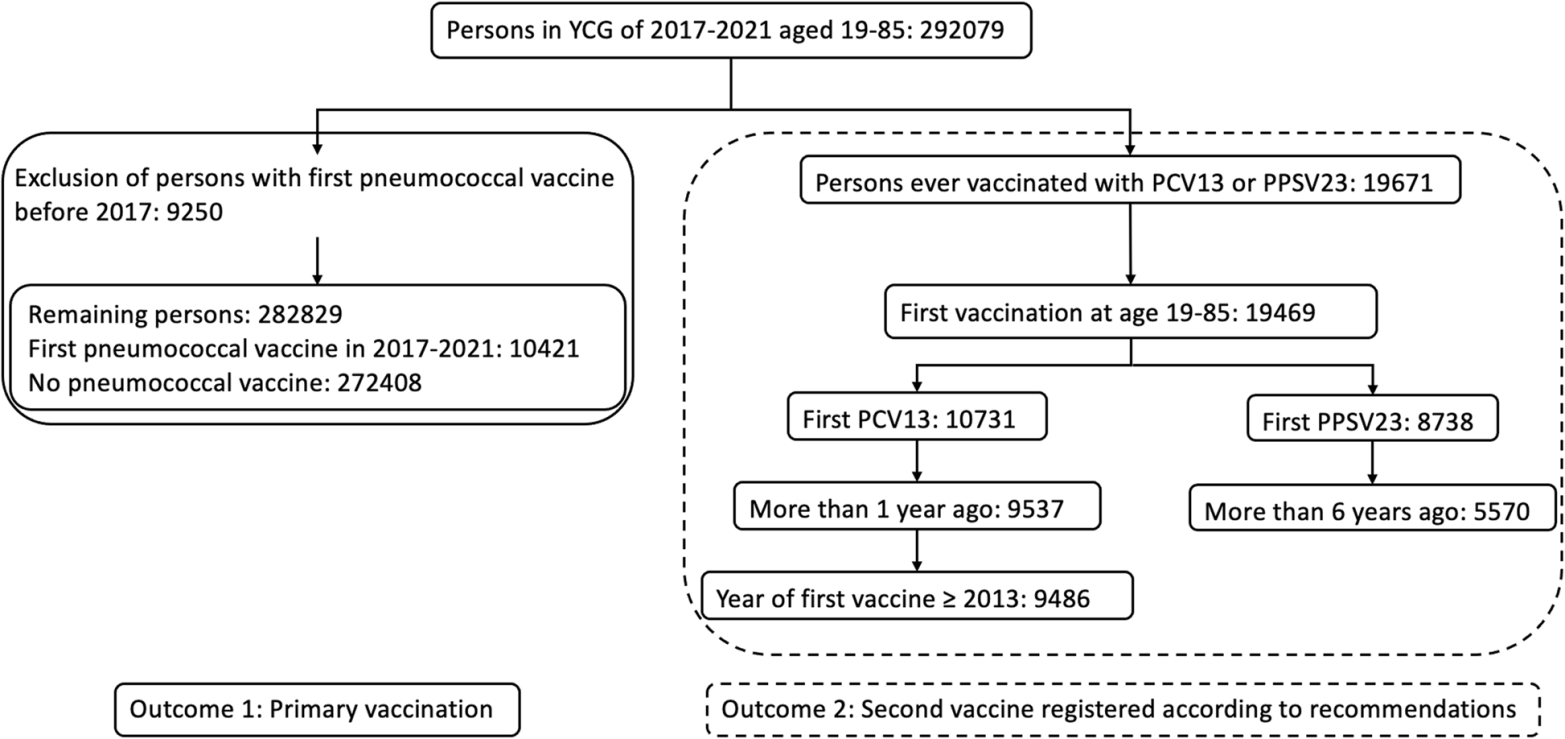
Flow chart of study population construction for modelling purposes

The flow chart in Fig. 1 shows the inclusion criteria to construct the study populations for investigation of the effect of an individual’s characteristics on primary vaccination and on schedule adherence. The population structure in terms of counts and proportions per target group and person characteristic for the outcomes of primary vaccination and of second vaccination adherence are given in Additional file 4.

### Pneumococcal vaccination coverage and schedule adherence

#### Seasonal trend in parallel with influenza vaccination

**Fig. 2:**
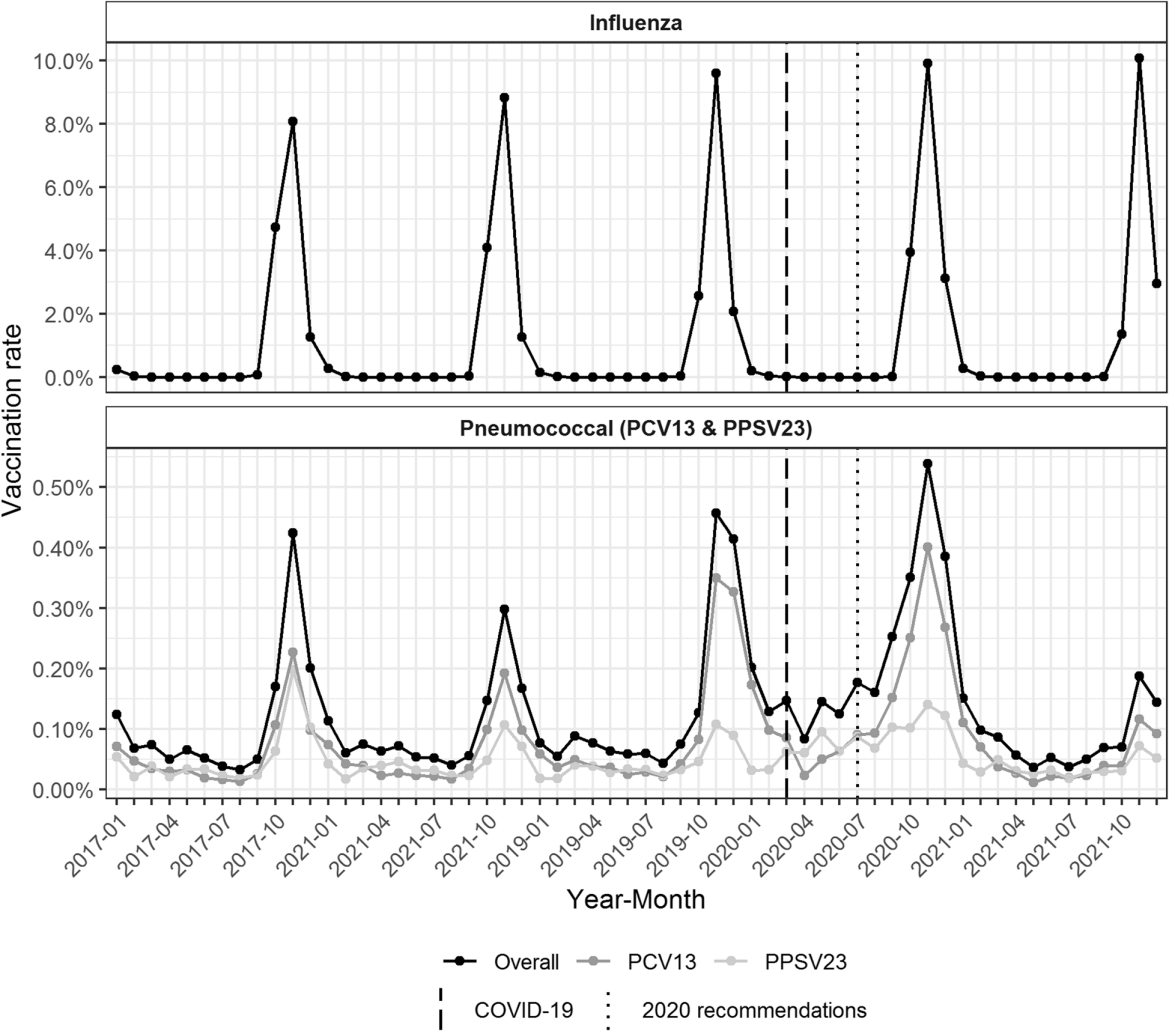
Evolution of influenza and pneumococcal monthly vaccination rates over time. Although pneumococcal vaccination is not a yearly or seasonal vaccine, it is administered in parallel with influenza vaccination

Adult pneumococcal vaccination displayed a seasonal trend that coincided with the seasonal influenza vaccination campaign, both of which reached their maximum rates around November (Fig. 2). At the peak, approximately one-tenth of the persons in the YCG received an influenza vaccination. Figure 2 indicates the start of the Belgian COVID-19 epidemic in March 2020 and the introduction of the new 2020 pneumococcal vaccination recommendations in July 2020. A slight but steady increase in the pneumococcal vaccination rate was noted during the first seven months after the start of the pandemic. Additionally, the monthly pneumococcal vaccination rate in the period of October-December 2021 was remarkably lower than that in previous years. Compared to November 2020, the pneumococcal vaccination rate in In November 2021 decreased from 0.54% to 0.19% (from 0.40% to 0.12% for PCV13 and 0.14% to 0.07% for PPSV23).

#### Vaccination coverage in Flanders

**Fig. 3:**
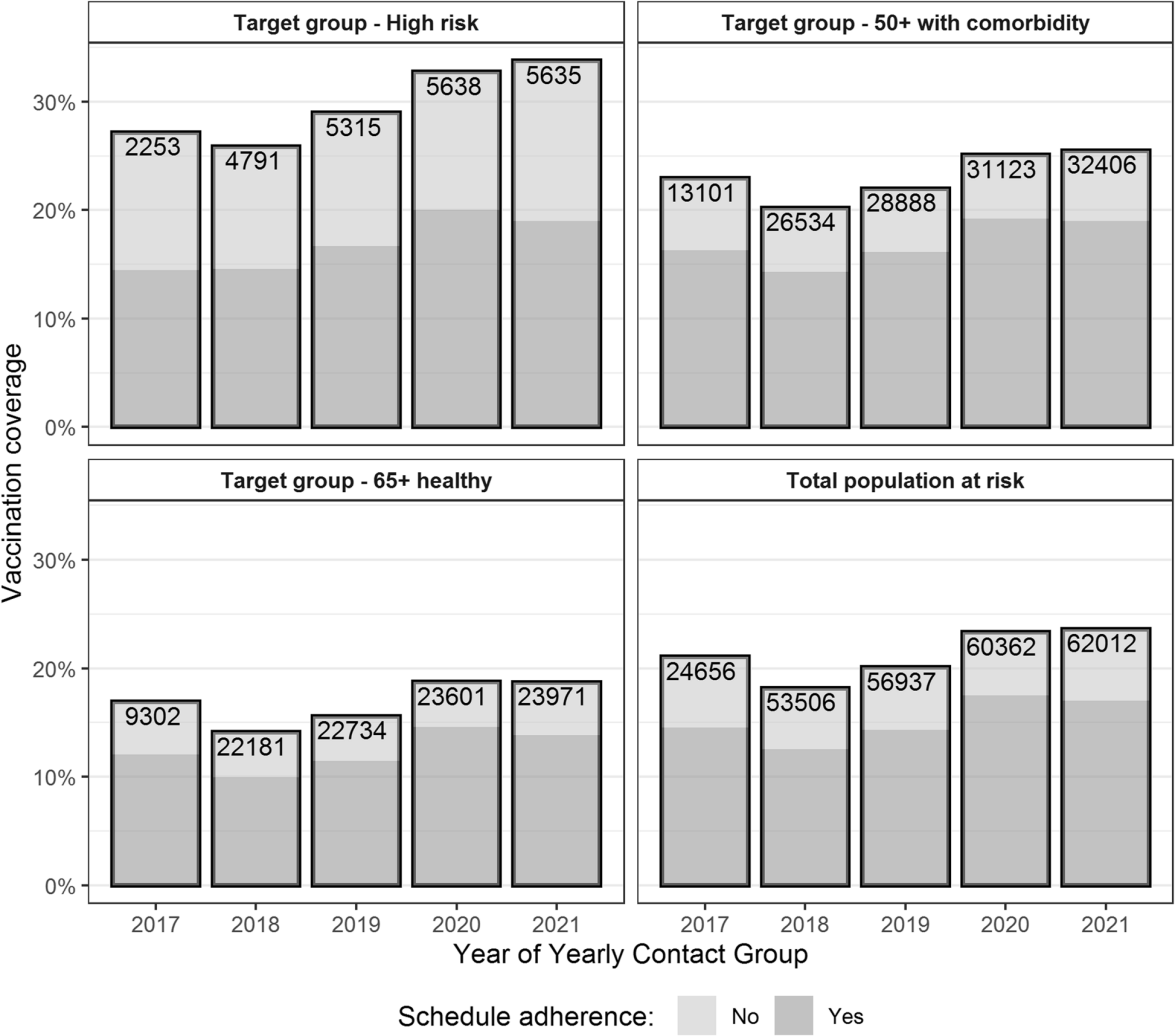
Vaccination coverage and adherence according to 2014 recommendations

**Fig. 4:**
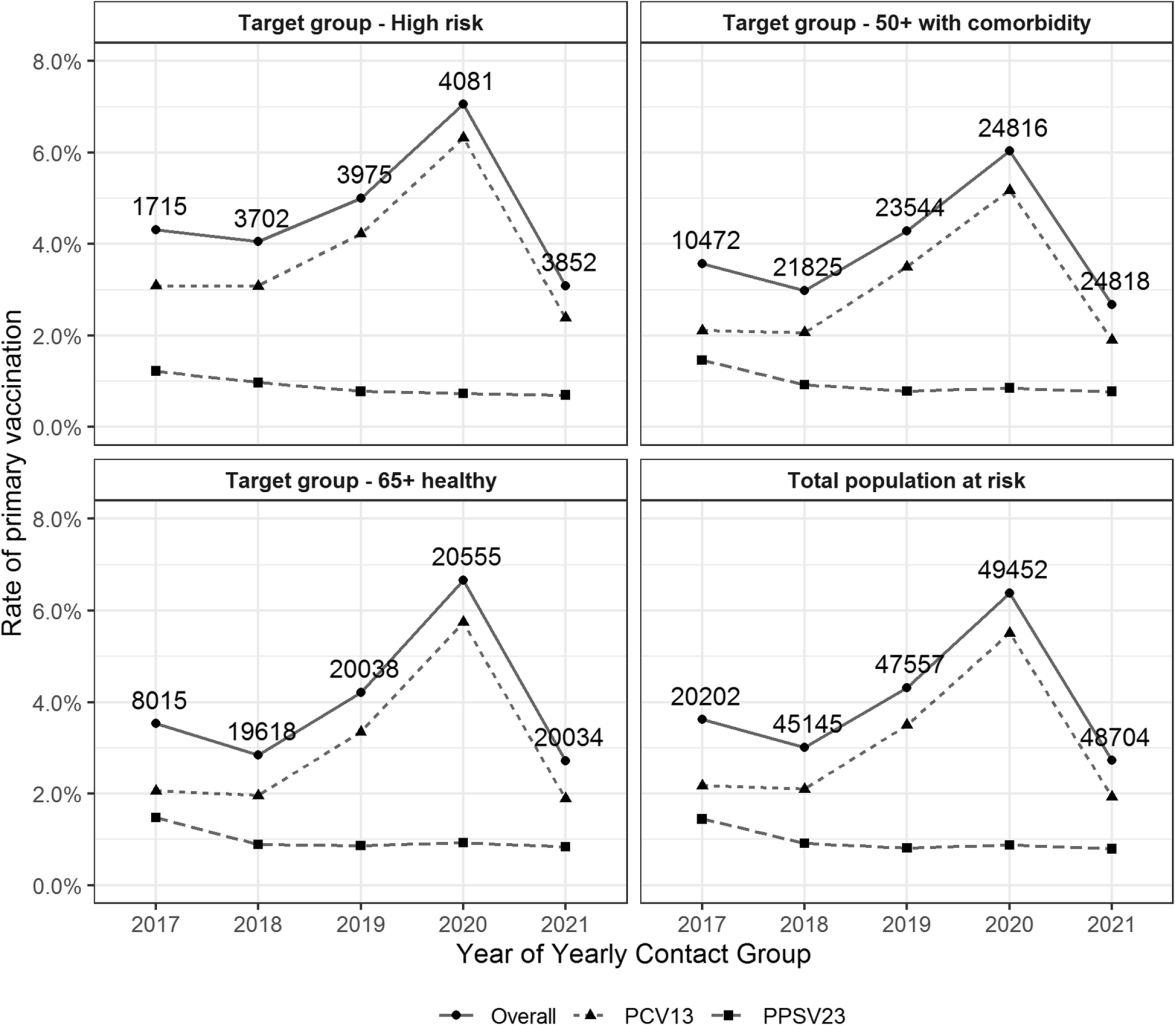
Yearly rate of primary pneumococcal vaccination per target group together with the denominator

Yearly pneumococcal vaccination coverage and the corresponding proportion of persons with a vaccination profile (based on the entire vaccination history) in adherence to the 2014 recommendations are displayed in Fig. 3 with the corresponding numbers presented in Additional file 5. Additionally, Fig. 4 shows the yearly rate of primary vaccination and the corresponding denominator, i.e., the total number of persons in the given target group of the YCG who were never vaccinated before.

The vaccination coverage exhibited a slight reduction from 2017 to 2018. However, from 2018 onwards, an increase in the proportion of immunized persons was observed. Vaccination coverage in the total population at risk increased from 18.2% in 2018 to 23.6% in 2021. Overall, a statistically significant increasing trend in the proportion of immunized persons in the population at risk was noted over the period of 2017–2021 (one-sided *p* value < 0.001). For each year in the period of 2017–2021, pneumococcal vaccination coverage (minimum %—maximum %) was highest in the high-risk group (25.9%-33.8%) followed by the 50+ with comorbidities (20.2%-25.5%) and 65+ healthy (14.1%-18.7%) groups.

The lowest proportion of persons with an adherent schedule was noted in the high-risk group, where only 55%-61% of the immunized persons had a schedule in adherence to the 2014 recommendations. However, in the other groups, this value was greater than 70%. The proportion of correctly vaccinated persons showed a statistically significant increasing trend over the period of 2017–2021 (one-sided Cochran-Armitage *p* value = 0.0001 <  < 0.05).

#### Determinants of primary vaccination (outcome 1)

**Fig. 5:**
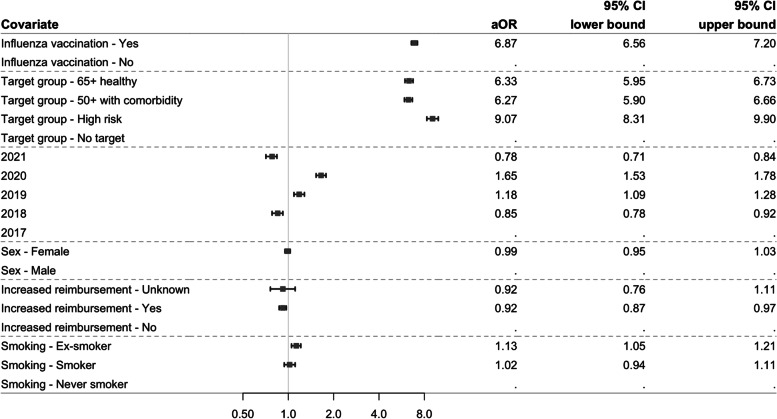
Forest plot showing the aOR (95% CI) for each person characteristic for primary vaccination

Figure 5 shows the aORs and associated 95% CIs of person characteristics on the probability of receiving a primary vaccination against pneumococcal disease. Primary vaccination is more likely for persons being vaccinated against influenza virus in the same year (6.87; 6.56–7.20), ex-smokers (1.13; 1.05–1.21), and belonging to any of the target groups where high-risk persons are most likely to receive primary pneumococcal vaccination (9.07; 8.31–9.90). Being in the yearly contact group of 2019 (1.18; 1.09–1.28) and 2020 (1.65; 1.53–1.78) increased the odds of primary vaccination. Persons with a lower socioeconomic status, i.e., receiving increased reimbursement, are less likely to receive primary vaccination (0.92; 0.87–0.97).

#### Determinants of adherence to second vaccine (outcome 2)

**Fig. 6:**
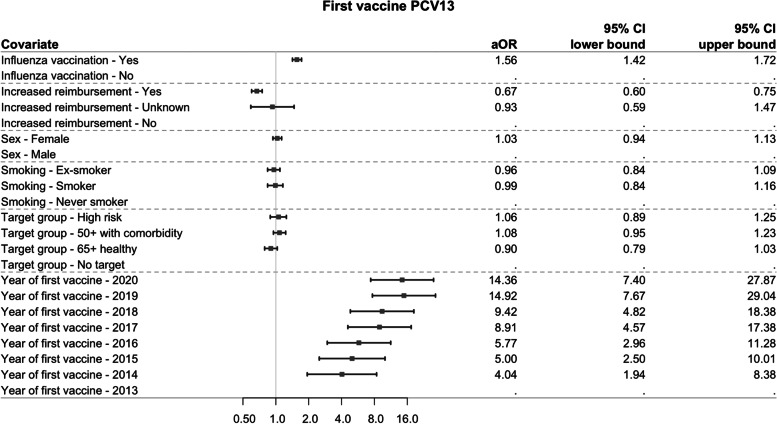
Forest plot of the aORs (95% CI) of adherence to second vaccine for each characteristic if the first vaccine was PCV13

**Fig. 7:**
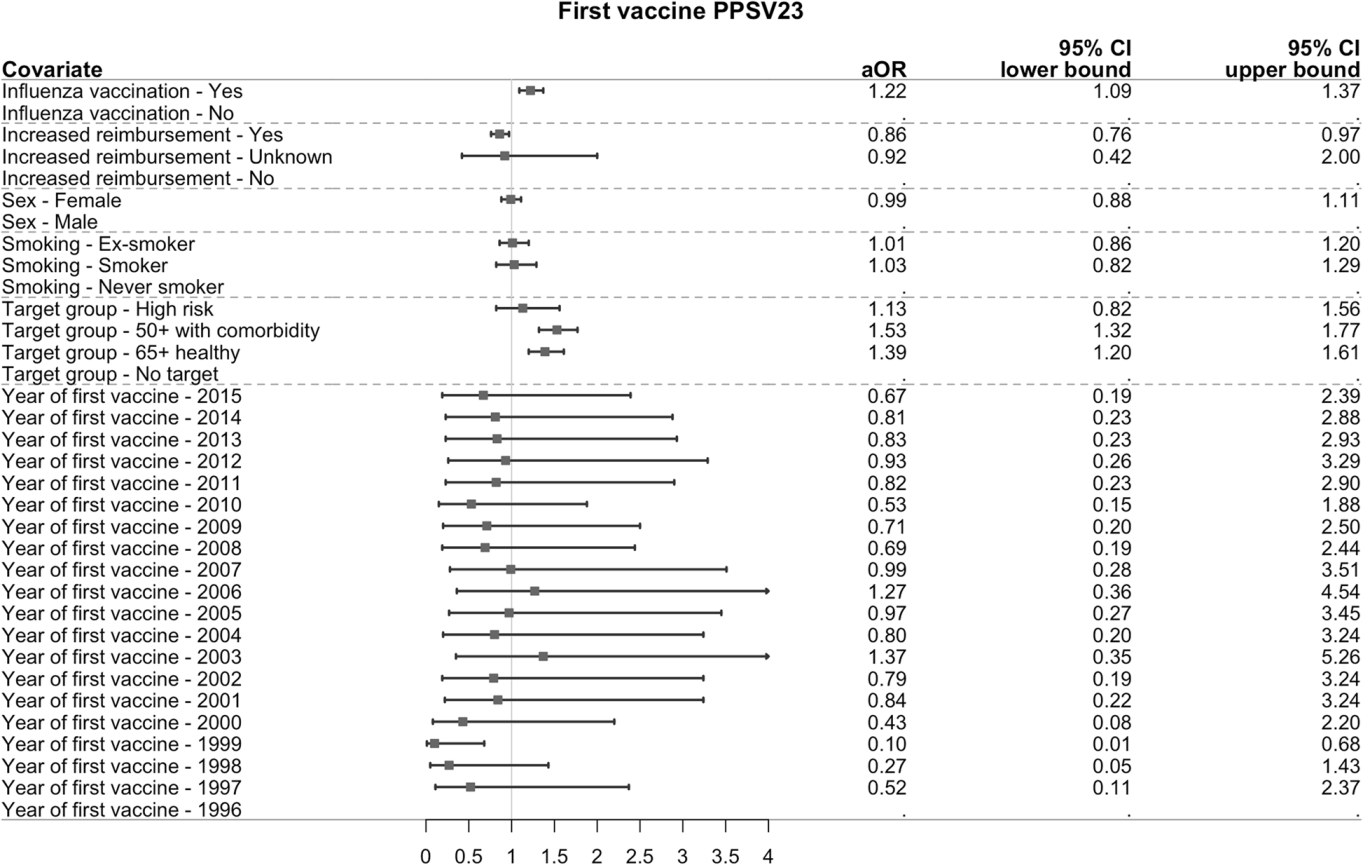
Forest plot of the aORs (95% CI) of adherence to second vaccine for each characteristic if the first vaccine was PPSV23

Figure 6 and Fig. 7 show the estimated aORs and their 95% CI for each individual characteristic and year of first vaccination with, respectively, PCV13 and PPSV23 as the first vaccine. Independent of the first vaccine, influenza vaccination in the year of first vaccination increased the odds of having a second vaccine in line with the recommendations (1.56 (1.42–1.72) for PCV13 and 1.22 (1.09–1.37) for PPSV23 as the first vaccine), whereas lower socioeconomic status decreased the odds of adherence (0.67 (0.60–0.75) for PCV13 and 0.86 (0.76–0.97) for PPSV23 as the first vaccine). Additionally, for the group receiving PCV13 as the first vaccine with 2013 as the reference year, persons being more recently vaccinated for the first time had a higher odds of adherence to the second vaccine. Persons in the 65+ healthy or 50+ with comorbidities target groups were more likely to have an adherent second vaccine if PPSV23 was the first vaccine.

## Discussion

### Main findings

Pneumococcal vaccination in Flanders occurs in parallel with seasonal influenza vaccination with a peak in November. The coverage in the population at risk only slightly increased from 2018 to 2021 to approximately 25%. The vaccination schedule adherence was lower in the high-risk adults with just over half of the population having a vaccination history according to the recommendations. In contrast, more than 70% of the lower-risk groups had an adherent schedule.

Adults receiving an influenza vaccination were more likely to receive a primary pneumococcal vaccine and to receive a second vaccine according to the recommendations compared to those without. Being an ex-smoker and belonging to the yearly contact group of 2019 and 2020 significantly increased the odds of primary vaccination. Having a lower socioeconomic background, approximated by increased reimbursement of healthcare, was detrimental for both the odds of primary vaccination and schedule adherence.

### Strengths and limitations

The main strength of this study is the inclusion of a large study population that is representative of the general Flemish population [17], currently approximating 5–6% of the population in Flanders. Furthermore, in addition to the availability of information about a proxy for socioeconomic status, influenza vaccination status and comorbidities, we could include smoking status using a longitudinal imputation method. To our knowledge, this study is one of the very few investigating the adherence to the recommended adult pneumococcal vaccination schedules and the association with an individual’s characteristics. Hence, our results provide useful insights into the association between certain population characteristics and pneumococcal vaccination status, including adherence to the rather complex recommended vaccination schedules.

Important limitations include the fact that only registered vaccines were included in this study. Most likely, all administered vaccines are not being registered (e.g., vaccines administered outside the family practice), which might result in an underestimation of the true vaccination coverage. Furthermore, due to the transition of the INTEGO network, discontinuity was noted in 2018 (i.e., the year of network expansion), which resulted in a doubling of the number of participants and the corresponding sudden reduction in vaccination coverage.

### Comparison to the literature

#### Seasonal trend in parallel with influenza vaccination

The monthly vaccination rate showed that the pneumococcal vaccination coincided with the seasonal influenza vaccination. The elevated pneumococcal vaccination rates in the second half of 2020, especially during the fall, might be a response to the COVID-19 pandemic in Belgium given that its Superior Health Council recommended pneumococcal vaccination to reduce the burden on hospitals due to these severe bacterial infections [10]. Furthermore, the seasonal peak rate of pneumococcal vaccines at the end of the year was much lower in 2021 compared to previous years. In November 2021, the pneumococcal vaccination rates decreased from approximately 0.54% to 0.19% compared to November 2020. Sales figures of pneumococcal vaccines of MSD (Merck Sharp & Dohme LLC, a subsidiary of Merck & Co., Inc., Rahway, NJ, USA) showed a total market decrease of 39% for 2021 compared to 2020 [20, 21], which might be associated with the start of the COVID-19 booster vaccination campaign in Belgium [22].

#### Pneumococcal vaccination coverage and schedule adherence

With a pneumococcal vaccination coverage of approximately 23% in 2021, Belgium performs worse than many European countries. Data from several countries showed that the implementation of a government-funded program leads to increased pneumococcal vaccination coverage rates; recent examples include the Netherlands (73% coverage of adults aged 73–79 in 2020) [23] and Denmark (59% coverage of 65 + adults as of October 2020) [24-26]. Comparable vaccination coverage is observed in Germany, where they report a pneumococcal vaccination coverage of 19% in the population older than 60 years in the first quarter of 2020 [27]. However, a call from Federal Minister of Health on March 9^th^, 2020 to promote vaccination of adults over 60 years old led to a four-fold increase in the number of pneumococcal vaccine prescriptions [28].

The pneumococcal vaccination guidelines differ between European countries in terms of age and/or risk recommendations as well as vaccine sequence and timing [5-7]. Our results showed a prevalence of schedule adherence of approximately 70% in the population at risk, with approximately 55% of the high-risk population and greater than 70% of the 65 + healthy and 50 + with comorbidity groups having an vaccination schedule in adherence to the recommendations. These numbers are higher than the 41% (*N* = 2,160) of persons with an adherent schedule in the period 2014–2017 reported by Belgian pharmacists [14]. The few studies investigating adherence to pneumococcal vaccination guidelines reported lower adherence proportions compared to our results. In Germany, only 4% of the primary vaccinated immunocompromised individuals received an adherent sequential vaccination, and a study from the United States showed that only 2.2% of high-risk adults completed the recommended pneumococcal vaccination sequence after six years of follow-up [13, 29].

Primary pneumococcal vaccination and an adherent second vaccination were more likely for individuals receiving influenza vaccination and less likely for persons with a lower socioeconomic status. Furthermore, higher risk groups and ex-smokers were more likely to receive primary pneumococcal vaccination. Consistent with these results, several studies across multiple countries found a strong correlation between influenza and pneumococcal vaccination coverage [30-32]. Although our data did not provide evidence for a significant association between current smokers and pneumococcal vaccination, other papers reported lower pneumococcal vaccination rates and stronger negative attitudes towards vaccination of current smokers [33, 34]. Similar to that observed, vaccination rates were higher in adults with comorbidities (or higher risk groups) [35, 36]. Lower socioeconomic status is often linked to lower awareness of vaccination and reduced vaccination coverage, which supports our finding of a lowered odds of primary vaccination and schedule adherence [37, 38]. Low socioeconomic status based on household income [38], education and absence of insurance coverage [39] was associated with lower uptake of pneumococcal vaccines. Persons with a household income in the lowest decile were 40% less likely to be vaccinated against pneumococcal disease than those with an income in the highest decile [38].

A study from 2011 reported higher pneumococcal vaccination coverage in European countries with public reimbursement and age-based recommendations [40]. Recent numbers show a similar impact of publicly reimbursed pneumococcal vaccines. In Catalonia, where PPSV23 is, similar to the rest of Spain, publicly funded for older people (≥ 65 years) and 19–65 years old with certain at-risk conditions and where PCV13 is only funded for mainly immunocompromised persons, pneumococcal vaccination coverage of the corresponding target populations in 2017 was 52.8% for PPSV23 but only 3.3% for PCV13 [41]. The highest reported pneumococcal vaccination coverage was found in England with 70.6% of adults aged 65 years and older ever being vaccinated since the start of the program in 2003 up to and including March 2021 [42]. Other countries that reimburse pneumococcal vaccination include France, where both PCV13 and PPSV23 vaccines are reimbursed up to 65% for certain risk groups such as immunocompromised persons and persons with comorbidities [43, 44]. Sweden will introduce a new national vaccination program in the autumn of 2022 that will offer pneumococcal vaccination to persons at risk for pneumococcal infection and persons aged 75 years or older [45, 46]. Belgium is one of few countries in Europe where pneumococcal vaccination is not publicly reimbursed, leading to inequity issues [5, 8].

The recommended pneumococcal vaccination schedules were introduced to ensure an optimal protection of risk groups against pneumococcal diseases. This notion is supported by several studies that found pneumococcal vaccination to be protective against invasive pneumococcal disease [47, 48]. Another study performed using the INTEGO database showed a protective effect of pneumococcal vaccination for severe LRTIs [12]. Our conclusions emphasize the importance of introducing a publicly funded program for adult pneumococcal vaccination in Belgium to ensure equity in access, to increase vaccination coverage and to achieve protection against pneumococcal diseases for the targeted population.

## Conclusions

General practice data from Flanders showed that pneumococcal vaccination coverage of target groups is slowly increasing. Pneumococcal vaccination rates exhibit seasonal peaks in parallel with seasonal influenza vaccination campaigns. Adults with poor socioeconomic status are less likely to receive primary pneumococcal vaccination and a second vaccination in adherence to the recommendations. These findings demonstrate the need for a publicly funded program in Belgium to ensure equitable access and to optimize the benefits of current recommendations for the target population.

## Supplementary Information

**Additional file 1.** ICPC-2, ICD-10, ATC codes used to identify comorbidities.

**Additional file 2.** Multiple imputation method and Rubin’s rules.

**Additional file 3.** Proportion of adults at risk in 2019 per comorbidity and age group.

**Additional file 4.** Population structure for the two outcomes: primary vaccination and adherent second vaccination.

**Additional file 5.** Pneumococcal vaccination coverage and adherence to the 2014 recommendations.

**Additional file 6.** Adjusted odds ratios of individual characteristics for the two outcomes.

## Data Availability

The datasets supporting the conclusions of this article are included within the article and its Additional files. The datasets generated and/or analysed during the current study are not publicly available due confidentiality but are available in aggregated format from the corresponding author on reasonable request.

## Abbreviations

CI: Confidence Interval
LRTI: Lower Respiratory Tract Infection
MSD: Merck Sharp & Dohme LLC, a subsidiary of Merck & Co., Inc., Rahway, NJ, USA
(a)OR: (Adjusted) Odds Ratio
PCV13: Pneumococcal Conjugate Vaccine 13-valent
PPSV23: Pneumococcal Polysaccharide Vaccine 23-valent
Q1: First Quartile
Q3: Third Quartile
SP: Streptococcus Pneumoniae
YCG: Yearly Contact Group

## References

[1] Torres A, Peetermans WE, Viegi G (2013). Risk factors for community-acquired pneumonia in adults in Europe: a literature review. Thorax.

[2] Rozenbaum MH, Pechlivanoglou P, van der Werf TS (2013). The role of Streptococcus pneumoniae in community-acquired pneumonia among adults in Europe: a meta-analysis. Eur J Clin Microbiol Infect Dis.

[3] Business Wire. European Commission Approves Merck’s VAXNEUVANCE^TM^ (Pneumococcal 15-Valent Conjugate Vaccine) for Individuals 18 Years of Age and Older. 2021. Available from: https://www.businesswire.com/news/home/20211215005345/en/ [Last accessed: 6/6/2022].

[4] Business Wire. European Medicines Agency Approves Pfizer’s 20-Valent Pneumococcal Conjugate Vaccine Against Invasive Pneumococcal Disease and Pneumonia in Adults. 2022. Available from: https://www.businesswire.com/news/home/20220215005838/en/ [Last accessed: 6/6/2022].

[5] Castiglia P (2014). Recommendations for pneumococcal immunization outside routine childhood immunization programs in Western Europe. Adv Ther.

[6] Bonnave C, Mertens D, Peetermans W (2019). Adult vaccination for pneumococcal disease: a comparison of the national guidelines in Europe. Eur J Clin Microbiol Infect Dis.

[7] European Centre for Disease Prevention and Control (ECDC). Pneumococcal Disease: Recommended Vaccinations. 2022. Available from: https://vaccine-schedule.ecdc.europa.eu/Scheduler/ByDisease?SelectedDiseaseId=25&SelectedCountryIdByDisease=-1 [Last accessed: 6/6/2022].

[8] Blommaert A, Hanquet G, Willem L, Theeten H, Thiry N, Bilcke J, et al. Use of pneumococcal vaccines in the elderly: an economic evaluation – synthesis. Health Technology Assessment (HTA) Brussels: Belgian Health Care Knowledge Centre (KCE); 2016. KCE Reports 274Cs. D/2016/10.273/78.

[9] Hoge Gezondheidsraad. Vaccinatie tegen pneumokokken (volwassenen). Brussel: HGR; 2014. Advies nr. 9210.

[10] Hoge Gezondheidsraad. Vaccinatie tegen pneumokokken (volwassenen). Brussel: HGR; 2020. Advies nr. 9562.

[11] Hoge Gezondheidsraad. Vaccinatie tegen pneumokokken (volwassenen). Brussel: HGR; 2022. Advies nr. 9674.

[12] Mamouris P, Henrard S, Molenberghs G (2022). Pneumococcal vaccination prevented severe LRTIs in adults: a causal inference framework applied in registry data. J Clin Epidemiol.

[13] Deb A, Mohanty S, Ou W (2021). Pneumococcal vaccination coverage among adults aged 19 to 64 years with immuno-compromising conditions, cerebrospinal fluid (CSF) leaks, or cochlear implants in the US. Expert Rev Vaccines.

[14] KOVAG. Vaccineren tegen pneumokokken: we zijn er nog niet! 2018. Available from: https://www.kovag.be/tarifering_artsen/uploads/documentenbank/b7b8db294e42c7f249628f3fca14c087.pdf [Last accessed: 9/13/2022].

[15] De Burghgraeve T, Henrard S, Verboven B (2021). The incidence of lower respiratory tract infections and pneumococcal vaccination status in adults in flemish primary care. Acta Clin Belg.

[16] Fischer F, Lange K, Klose K (2016). Barriers and Strategies in Guideline Implementation—A Scoping Review. Healthcare.

[17] Truyers C, Goderis G, Dewitte H (2014). The Intego database: background, methods and basic results of a Flemish general practice-based continuous morbidity registration project. BMC Med Inform Decis Mak.

[18] Delvaux N, Aertgeerts B, van Bussel JC (2018). Health data for research through a nationwide privacy-proof system in belgium: design and implementation. JMIR Med Inform.

[19] R Core Team. R: a language and environment for statistical computing. Vienna: R Foundation for Statistical Computing; 2020. https://www.R-project.org/.

[20] IQVIA. 2020 ACTS annual report: statistical quality assurance, applied to IQVIA’s information offerings. 2021. Available from: https://www.iqvia.com/-/media/iqvia/pdfs/library/publications/2020-acts-annual-report.pdf. Accessed 6 Jan 2023.

[21] IQVIA. 2021 ACTS annual report: statistical quality assurance, applied to IQVIA’s information offerings. 2022. Available from: https://www.iqvia.com/-/media/iqvia/pdfs/library/publications/2021-acts-annual-report.pdf. Accessed 6 Jan 2023.

[22] Superior Health Council. Booster vaccination against COVID-19 for the general population. Brussels: SHC; 2021. Report 9683.

[23] RIVM. Pneumokokkenprik Voor Volwassenen - Vaccinatiegraad. 2022. Available from: https://www.rivm.nl/pneumokokken/pneumokokkenprik/vaccinatiegraad [Last accessed: 6/2/2022].

[24] Valentiner-Branth P. No 14/16 - 2020: Pneumococcal Vaccination Programme for Persons Aged 65 Years or More and for Risk Groups. Statens Serum Institut. 2020. Available from: https://en.ssi.dk/news/epi-news/2020/no-14---2020 [Last accessed: 6/6/2022].

[25] Valentiner-Branth P. No 23 - 2020: Everyone above 65 Years of Age Will Be Offered Pneumococcal Vaccination. Statens Serum Institut. 2020. Available from: https://en.ssi.dk/news/epi-news/2020/no-23---2020 [Last accessed: 6/6/2022].

[26] Statens Serum Institut. No 44/45 - 2020: Update on Influenza and Pneumococcal Vaccination. 2020. Available from: https://en.ssi.dk/news/epi-news/2020/no-44-45---2020 [Last accessed: 6/6/2022].

[27] Standing Vaccination Committee (STIKO) at the Robert Koch Institute (2020). Impfquoten bei Erwachsenen in Deutschland, STIKO: Bestätigung der Pneumokokken-Impfempfehlung. Epidemiologisches Bulletin.

[28] Wallenfels M (2020). Pneumokokken-Vakzine: Corona führt zu hoher Impfbereitschaft. CME Berl Ger.

[29] Sprenger R, Häckl D, Kossack N (2022). Pneumococcal vaccination rates in immunocompromised patients in Germany: A retrospective cohort study to assess sequential vaccination rates and changes over time. PLOS ONE.

[30] Heins M, Hooiveld M, Korevaar J. Monitor Vaccinatiegraad Nationaal Programma Pneumokokkenvaccinatie Volwassenen (NPPV) 2020. Utrecht: NIVEL; 2021.

[31] Miller LS, Kourbatova EV, Goodman S (2005). Brief report: risk factors for pneumococcal vaccine refusal in adults: a casecontrol study. J Gen Intern Med.

[32] Sakamoto A, Chanyasanha C, Sujirarat D (2018). Factors associated with pneumococcal vaccination in elderly people: a cross-sectional study among elderly club members in Miyakonojo City, Japan. BMC Public Health.

[33] Pearson WS, Dube SR, Ford ES (2009). Influenza and pneumococcal vaccination rates among smokers: data from the 2006 Behavioral Risk Factor Surveillance System. Prev Med.

[34] Jackson SE, Paul E, Brown J (2021). Negative Vaccine Attitudes and Intentions to Vaccinate Against Covid-19 in Relation to Smoking Status: A Population Survey of UK Adults. Nicotine Tob Res Off J Soc Res Nicotine Tob.

[35] Carreño-Ibáñez LV, Esteban-Vasallo MD, Domínguez-Berjón MF (2015). Coverage of and factors associated with pneumococcal vaccination in chronic obstructive pulmonary disease. Int J Tuberc Lung Dis.

[36] Aka Aktürk Ü, Görek Dilektaşlı A, Şengül A (2017). Influenza and Pneumonia Vaccination Rates and Factors Affecting Vaccination among Patients with Chronic Obstructive Pulmonary Disease. Balk Med J.

[37] Baig SA, Hassan M, Ahmed SM (2014). A cross-sectional study to investigate pneumococcal vaccination in the elderly in a low income county: Patient knowledge, awareness, and attitudes of vaccination and prevalence rates by socioeconomic status. Hum Vaccines Immunother.

[38] McLaughlin JM, Swerdlow DL, Khan F (2019). Disparities in uptake of 13-valent pneumococcal conjugate vaccine among older adults in the United States. Hum Vaccines Immunother.

[39] Tsachouridou O, Georgiou A, Naoum S (2019). Factors associated with poor adherence to vaccination against hepatitis viruses, streptococcus pneumoniae and seasonal influenza in HIV-infected adults. Hum Vaccines Immunother.

[40] Fedson DS, Nicolas-Spony L, Klemets P (2011). Pneumococcal polysaccharide vaccination for adults: new perspectives for Europe. Expert Rev Vaccines.

[41] Vila-Córcoles A, Ochoa-Gondar O, de Diego C (2019). Pneumococcal vaccination coverages by age, sex and specific underlying risk conditions among middle-aged and older adults in Catalonia, Spain, 2017. Eurosurveillance.

[42] UK Health Security Agency. Pneumococcal Polysaccharide Vaccine (PPV) coverage report, England, April 2020 to March 2021. 2021. Available from: https://www.gov.uk/government/publications/pneumococcal-polysaccharide-vaccine-ppv-vaccine-coverage-estimates. Accessed 6 Mar 2022.

[43] MesVaccins.net. PREVENAR 13. 2021. Available from: http://www.mesvaccins.net/web/vaccines/123-prevenar-13 [Last accessed: 6/9/2022].

[44] MesVaccins.net. PNEUMOVAX. 2022. Available from: http://www.mesvaccins.net/web/vaccines/336-pneumovax [Last accessed: 6/9/2022].

[45] Public Health Agency of Sweden. Pneumokockvaccination som ett särskilt vaccinationsprogram för personer 75 år och äldre. 2021. Available from: https://www.folkhalsomyndigheten.se/publicerat-material/publikationsarkiv/p/pneumokockvaccination-som-ett-sarskilt-vaccinationsprogram-for-personer-75-ar-och-aldre/ [Last accessed: 6/3/2022].

[46] Public Health Agency of Sweden. Vaccination Programmes. 2022. Available from: https://www.folkhalsomyndigheten.se/the-public-health-agency-of-sweden/communicable-disease-control/vaccinations/vaccination-programmes/ [Last accessed: 6/3/2022].

[47] Moberley S, Holden J, Tatham DP (2013). Vaccines for preventing pneumococcal infection in adults. Cochrane Database Syst Rev.

[48] Perniciaro S, van der Linden M (2021). Pneumococcal vaccine uptake and vaccine effectiveness in older adults with invasive pneumococcal disease in Germany: A retrospective cohort study. Lancet Reg Health – Eur.

