# Supplementary material for: Pneumococcal vaccination coverage and adherence to recommended dosing schedules in adults: a repeated cross-sectional study of the INTEGO morbidity registry
Source: BMC Public Health. 2023 Jun 7;23:1104. doi: 10.1186/s12889-023-15939-7 (PMC10245355; doi:10.1186/s12889-023-15939-7)
Supplement: Supplementary file 1 — Additional file 1. ICPC-2, ICD-10, ATC codes used to identify comorbidities. [file 12889_2023_15939_MOESM1_ESM.pdf]

ICPC-2, ICD-10 and ATC codes used to identify comorbidities

**ICPC-2 codes, ICD-10 codes and ATC codes used for comorbidities**		
High-risk persons	HIV AIDS (ICPC-2 B90)	Ever diagnosed before 31/12/YEAR
	Lymphoma (ICPC-2 B72)	Ever diagnosed before 31/12/YEAR
	Leukaemia (ICPC-2 B73)	Ever diagnosed before 31/12/YEAR
	Malignant neoplasm (ICPC-2 B74)	Ever diagnosed before 31/12/YEAR
	Asplenia (ICD-10 D73.0)	Ever diagnosed before 31/12/YEAR
	Sickle-cell disease (ICPC-2 B78)	Ever diagnosed before 31/12/YEAR
	Cerebrospinal fluid leak (ICD-10 G96.0)	01/01/YEAR – 31/12/YEAR
	Cochlear implant (ICD-10 Z96.2)	Ever diagnosed before 31/12/YEAR
	Immunosuppressants (ATC L04)	in YEAR or YEAR-1
	Glucocorticoids (ATC H02AB)	at least 2x in YEAR
Medium-risk persons	Chronic bronchitis (ICPC-2 R79)	Ever diagnosed before 31/12/YEAR
	Congenital respiratory anomaly (ICPC-2 R89)	Ever diagnosed before 31/12/YEAR
	COPD (ICPC-2 R95)	Ever diagnosed before 31/12/YEAR
	Asthma (ICPC-2 R96)	Ever diagnosed before 31/12/YEAR
	Congenital cardiovascular anomaly (ICPC-2 K73)	Ever diagnosed before 31/12/YEAR
	Angina pectoris (ICPC-2 K74)	Ever diagnosed before 31/12/YEAR
	Acute myocardial infarction (ICPC-2 K75)	Ever diagnosed before 31/12/YEAR
	Chronic ischaemic heart disease (ICPC-2 K76)	Ever diagnosed before 31/12/YEAR
	Heart failure (ICPC-2 K77)	Ever diagnosed before 31/12/YEAR
	Atrial fibrillation (ICPC-2 K78)	Ever diagnosed before 31/12/YEAR
	Pulmonary heart disease (ICPC-2 K82)	Ever diagnosed before 31/12/YEAR
	Heart valve disease (ICPC-2 K83)	Ever diagnosed before 31/12/YEAR
	TIA (ICPC-2 K89)	Ever diagnosed before 31/12/YEAR
	CVA (ICPC-2 K90)	Ever diagnosed before 31/12/YEAR
	Peripheral arterial disease (ICPC-2 K92)	Ever diagnosed before 31/12/YEAR
	Chronic liver disease (ICPC-2 D97)	Ever diagnosed before 31/12/YEAR
	Chronic alcohol abuse (ICPC-2 P15)	Ever diagnosed before 31/12/YEAR
	Chronic kidney disease eGFR < 60 mL/min	1x <60 in YEAR-2 or YEAR-2
	Diabetes mellitus (ICPC-2 T89 or T90)*	Ever diagnosed before 31/12/YEAR
	Multiple sclerosis (ICPC-2 N86)*	Ever diagnosed before 31/12/YEAR
	Parkinson's disease (ICPC-2 N87)*	Ever diagnosed before 31/12/YEAR
	Muscular dystrophy (ICD-10 G71.0)*	Ever diagnosed before 31/12/YEAR
	Myasthenia gravis (ICD-10 G70.0)*	Ever diagnosed before 31/12/YEAR
	Lambert-Eaton syndrome (ICD-10 G73.1)*	Ever diagnosed before 31/12/YEAR
	Myopathy (ICD-10 G70, G71, G72, G73)*	Ever diagnosed before 31/12/YEAR
	Motor neuron disease (ICD-10 G12.2)*	Ever diagnosed before 31/12/YEAR
	Charcot-Marie-Tooth disease (ICD-10 G60.0)*	Ever diagnosed before 31/12/YEAR
Low-risk persons	All other persons in the yearly contact group of YEAR	

*: comorbidities included in 2020 recommendations

YEAR: the year for which the disease profile is constructed
