# Supplementary material for: Pneumococcal vaccination coverage and adherence to recommended dosing schedules in adults: a repeated cross-sectional study of the INTEGO morbidity registry
Source: BMC Public Health. 2023 Jun 7;23:1104. doi: 10.1186/s12889-023-15939-7 (PMC10245355; doi:10.1186/s12889-023-15939-7)
Supplement: Supplementary file 2 — Additional file 2. Multiple imputation method and Rubin’s rules. [file 12889_2023_15939_MOESM2_ESM.pdf]

Multiple imputation method and Rubin's rules

In the setting of longitudinal data, the temporal structure can be easily distorted, and complexities, such as collinearity, convergence and overfitting, frequently occur. In collaboration with Nassiri Vahid, Verbeeke Geert, Vaes Bert and Molenberghs Geert, Mamouris Pavlos developed a 3-stage methodology that imputes longitudinal categorical data in a flexible manner by separating the standard imputation approach with a modelling phase of a joint Markov model that accommodates forward, backward, and intermittent probabilities. Using this method, smoking, alcohol, and other categorical covariates can be imputed efficiently, avoiding the complexities mentioned above. At the time of writing, this work is under review and not yet published. We used their methodology to longitudinally impute the smoking covariate 20 times, resulting in 20 datasets. We then performed the analysis on each imputed dataset and obtained a single set of (a)OR estimates and CIs by pooling the results using Rubin's rules[51]. We elaborate the rules in the next paragraph.

Suppose $\hat{\beta}_i$ denotes the estimate of a parameter β, e.g., a regression coefficient, from the kth dataset ($k = 1 \dots m$, in our case $m = 20$) with a variance $\widehat{U}_k$. The pooled point estimate of β is then given as follows:

$$\bar{\beta} = \frac{1}{m}\sum_{k=1}^{m} \hat{\beta}_k.$$

The variance of $\bar{\beta}$, which is denoted as T, is the weighted sum of the within-imputation variance ($\overline{U}$) and the between-imputation variance (B):

$$\overline{U} = \frac{1}{m}\sum_{k=1}^{m} \widehat{U}_k,$$

$$B = \frac{1}{m-1}\sum_{k=1}^{m}(\hat{\beta}_k - \bar{\beta})^2,$$

$$T = \overline{U} + \left(1 + \frac{1}{m}\right) B.$$

The statistic $\frac{Q-\bar{Q}}{\sqrt{T}}$ approximately follows a t distribution. The degrees of freedom are computed as follows:

$$r = \frac{\left(1+\frac{1}{m}\right)B}{\bar{U}},$$

$$v_m = (m-1)(1+\frac{1}{r})^2,$$

$$v_m^* = [\frac{1}{v_m} + \frac{1}{\frac{(1-\gamma)v_0(v_0+1)}{v_0+3}}]^{-1},$$

where r is the relative increase in variance due to missing data, i.e., the adjusted between-imputation variance standardized by the within-imputation variance; $\gamma = (1+1/m)\,B/T$ and v_0 are the degrees of freedom in the complete data; and v_m^* is a correction of v_m if v_0 is small and there is a limited amount of missing data. The 95% CI can then be computed using the general formula:

$$\bar{\beta} \pm t_{df,1-\alpha/2} * SE_{pooled},$$

with $df = v_m$ and $SE_{pooled} = \sqrt{T}$.
